# Isolated polyhydramnios in the third trimester or polyhydramnios secondary to late-onset gestational diabetes: is it worth distinguishing?

**DOI:** 10.1590/1806-9282.20231390

**Published:** 2024-06-17

**Authors:** Sadullah Özkan, Murat Levent Dereli, Sadun Sucu, Erol Nadi Varlı, Arife Akay, Safiye Elif Uzlu, Ali Turhan Çağlar, Yaprak Engin-Ustun

**Affiliations:** 1 Etlik Lady Zübeyde Maternity and Women's Health Teaching and Research Hospital, Department of Obstetrics and Gynaecology – Ankara, Turkey.; 2 Etlik Lady Zübeyde Maternity and Women's Health Teaching and Research Hospital, Department of Neonatology – Ankara, Turkey.

**Keywords:** Gestational diabetes mellitus, Polyhydramnios, Late-onset, Outcome, Oral glucose tolerance test

## Abstract

**OBJECTIVE:**

The aim of this study was to compare pregnancy outcomes of patients with polyhydramnios due to late-onset gestational diabetes mellitus and patients with isolated polyhydramnios.

**METHODS:**

Of the women who fully participated in prenatal examinations at Etlik Lady Zübeyde Hospital between January 1, 2018, and December 31, 2019, women with polyhydramnios of nonfetal–placental origin manifesting in the third trimester were retrospectively reviewed. Women with normal 75-g oral glucose tolerance test results between 24 and 28 weeks gestation who met the inclusion criteria were enrolled in the study and divided into two groups based on the results of rescreening with the 75-g oral glucose tolerance test for polyhydramnios in the third trimester: women with isolated polyhydramnios (group 1) and women with late-onset polyhydramnios due to gestational diabetes mellitus (group 2).

**RESULTS:**

There were a total of 295 participants, of whom 35 (11.8%) were diagnosed with polyhydramnios due to late-onset gestational diabetes mellitus. There were no differences in the main outcomes. Birthweight and gestational age at birth were identified as independent risk factors for predicting composite maternal outcome {[odds ratio (OR)=1.273, 95% confidence interval (CI) 1.063–1.524, p=0.009]} and composite neonatal outcome (OR=0.606, CI 0.494–0.744, p<0.001), respectively.

**CONCLUSION:**

Polyhydramnios in late pregnancy without evidence of pregnancy-related causes leading to polyhydramnios may be a sign of late-onset gestational diabetes mellitus in women with a normal prior oral glucose tolerance test. As pregnancy outcomes and management were indifferent, it does not seem necessary or useful to diagnose whether or not late-onset gestational diabetes mellitus is present.

## INTRODUCTION

Polyhydramnios, defined as an increase in amniotic fluid volume, occurs in 0.4–2.0% of all pregnancies^1^. While about 60% of cases are idiopathic, known causes include fetal anomalies leading to fetal dysphagia or increased fetal urination, fetal–placental disorders causing fetal anemia, and maternal diabetes^2,3^. Polyhydramnios in which no causes can be identified are called isolated polyhydramnios^2^.

Gestational diabetes mellitus (GDM) is one of the most common complications of pregnancy (4–16%), which is defined as a disorder of glucose metabolism that occurs or is first recognized during pregnancy^4^. A 75-g oral glucose tolerance test (OGTT) is the most widely used method for diagnosing GDM, although there are new promising methods such as measuring the fetal thymic–thoracic ratio or fetal thymus transverse diameter^5^. GDM increases the risk of severe pregnancy complications such as macrosomia, shoulder dystocia, cesarean section, wound infection, polyhydramnios, and neonatal hypoglycemia^6^. The underlying mechanism by which polyhydramnios develop in maternal diabetes is not fully understood. The highlighted pathophysiology is fetal polyuria caused by increased osmotic diuresis due to fetal hyperglycemia and increased accumulation of fluid from the chorioamniotic membranes due to higher glucose concentrations in the amniotic fluid^4^.

Despite normal screening tests at 24–28 weeks of gestation (WG), some physicians recommend retesting for late-onset GDM in the third trimester if macrosomia is suspected or polyhydramnios develops^7^. In addition, there are still many questions about the side effects and benefits of antihyperglycemic medications in pregnancy that need to be clarified. Besides, many studies have demonstrated the association between cause-specific polyhydramnios and adverse perinatal outcomes, whereas the association between isolated polyhydramnios and adverse maternal–neonatal outcomes is contradictory^8,9^. As there is a need for studies that bridge the gap between theory and practice regarding perinatal and maternal outcomes in such pregnancy populations, we conducted this study.

## METHODS

### Study design

Medical records of all women diagnosed with third-trimester polyhydramnios between January 1, 2018, and December 31, 2019, in the high-risk pregnancy department of Etlik Lady Zübeyde Maternity and Women's Health Education and Research Hospital, Ankara, Turkey, were retrospectively reviewed after the hospital's local medical research ethics committee approved the conduct, protocol, and procedures of the study (09/29-2020-15/08).

### Study population characteristics, patient selection, and definitions

Pregnant women with an abnormal 75-g OGTT performed at 24–28 WG of current pregnancy, metabolic disorders other than late-onset GDM, multiple pregnancies, and patients with missing data and pregnancies with fetal features such as congenital infections, malformations, neuromuscular disorders, anemia, and genetic abnormalities were excluded. During the study period, there were a total of 29,238 live singleton births. Of these, 562 (1.9%) were diagnosed with third trimester polyhydramnios. Finally, 295 women without fetal–placental pathology who met the study criteria were included. Of the 295 (100%) participants, 258 (88.2%) were diagnosed with isolated polyhydramnios (group 1), whereas 35 (11.8%) were diagnosed with polyhydramnios due to late-onset GDM (group 2).

The diagnosis of GDM was made using the International Association of Diabetes and Pregnancy Study Groups consensus panel criteria^10^. Gestational age was calculated from the first day of the last menstrual period and confirmed by sonographic dating. Polyhydramnios was identified with the same sonography system [GE Voluson 730 Expert System (General Electric Medical Systems, Milwaukee, WI, USA) with a 2–7-MHz abdominal transducer] and classified as mild, moderate, or severe based on sonographic measurement of the maximum vertical pocket (MVP) of the amniotic fluid of 8–11, 12–15, and ≥16 cm, respectively^8^.

### Data collection

All data were obtained from the hospital database and medical records and compared between the two groups.

No serious neonatal adverse events occurred in either group, including neonatal death, sepsis, meconium aspiration syndrome, intraventricular hemorrhage, and necrotizing enterocolitis. Due to low incidence of some neonatal and maternal complications, which led to underestimation of the difference between the two groups, we combined various components of neonatal and maternal outcomes into composite outcome measures to draw conclusions. In the present study, the composite neonatal outcome (CNO) consisted of at least one or more of the following: neonatal intensive care unit (NICU) admission, shoulder dystocia, birth asphyxia, APGAR scores (appearance, pulse, grimace, activity, and respiration) of less than 7 at 5 min, transient tachypnea of newborn, respiratory distress syndrome (RDS) need for mechanical ventilation and continuous positive airway pressure (CPAP) therapy, hypoglycemia, hyperbilirubinemia, premature rupture of membranes (PROM), cord prolapse, and abruptio placentae. On the other hand, the composite maternal outcome (CMO) consisted of at least one or more of the following adverse maternal complications, including third- or fourth-degree vaginal tears, postpartum hemorrhage (PPH) due to uterine atony, hypertensive disorders of pregnancy (HDP), postpartum endometritis, and wound infection.

### Statistical analysis

All statistical analyses were performed using the RStudio program (version 2021.09.4+403.pro3). Kolmogorov-Smirnov or Shapiro-Wilk tests were used to assess normality. Levene's test was used to assess homogeneity of variance. Descriptive analyses for normally distributed variables were presented using means and standard deviation. The independent-samples t-test was used to compare these parameters between groups. Descriptive analyses for the numerical data that were not normally distributed were performed using medians and quartiles (Q1–Q3). Mann-Whitney U tests were used to compare these parameters between groups. Descriptive analyses for the categorical variables were performed using frequency and percentage. Relationships between categorical variables were analyzed using the chi-square test or Fisher's exact test when expected cell counts were low. For multivariate analysis, the possible factors identified in the univariate analyses were entered into a binary logistic regression analysis to identify additional independent predictors of CMO and CNO. The Hosmer-Lemeshow goodness-of-fit statistic was used to assess model fit. A type I error level of 5% overall was used to derive statistical significance. A p-value of <0.05 was considered a statistically significant result.

## RESULTS

Demographic and clinical characteristics at the time of diagnosis are described in Table 1. Mean number of previous cesarean deliveries, previous miscarriages, amniotic fluid measurements, and mean fasting blood glucose levels were indifferent between the two groups, while mean values for age, body mass index (BMI), gravidity, and parity were significantly higher in group 2 (p=0.001, 0.005, 0.012, and 0.022, respectively). Moreover, there was no difference in the severity of polyhydramnios.

**Table 1:** Demographic and clinical characteristics of patients in the study groups at the time of diagnosis.

Variables		Group 1 (n=258)	Group 2 (n=35)	Total (n=293)	p-value
Age (years)		28 (24–32)	34 (26–37)	28 (24–33)	**0.001**
BMI (kg/m^2^)		29 (26–32)	32 (28–35)	29 (26–33)	**0.005**
Gravidity (number)		3 (2–3)	3 (2–4)	3 (2–4)	**0.012**
Parity (number)		1 (0–2)	2 (1–3)	1 (1–2)	**0.022**
Previous cesarean delivery		0 (0–0)	0 (0–1)	0 (0–0)	0.073
Miscarriage (number)		0 (0–0)	0 (0–1)	0 (0–0)	0.734
MVP of amniotic fluid (mm)		95 (87–109.2)	97 (85–110)	95 (87–110)	0.857
	Mild	227 (88%)	31 (88.6%)	258 (88.1%)	0.322
	Moderate	25 (9.7%)	2 (5.7%)	27 (9.2%)	
	Severe	6 (2.3%)	2 (5.7%)	8 (2.7%)	
Fasting blood glucose (g/dL)		80 (75–88)	79 (71–98)	79.5 (75–88.2)	0.931
Postprandial blood glucose (g/dL)		113.2±22.2	116.1±34.3	113.9±24.9	**0.013**
HbA1c (%)		5.1±0.4	5.9±0.8	5.3±0.6	**<0.001**

BMI: body mass index; HbA1c: glycated hemoglobin; MVP: maximum vertical pocket; PHA: polyhydramnios. Data are expressed as median (Q1–Q3), mean±standard deviation or number (percentage) where appropriate. A p<0.05 indicates a significant difference. Statistically significant p-values are indicated in bold.

An analysis of birth characteristics and early neonatal and maternal outcomes of the study groups is shown in Table 2. The rate of cesarean delivery was high in both groups, with no statistical difference between the two groups, and the most common indication was labor with a previous cesarean scar. Birth characteristics, including mean number of preterm deliveries, birthweight, APGAR scores at the first and fifth minutes, female gender, NICU admission, hyperbilirubinemia, RDS, PROM, HDP, and PPH, were indifferent. Gestational age at birth (GAB) was significantly higher in group 1, while hypoglycemia was more frequent in group 2 (p<0.001 and p=0.014, respectively). No significant differences were found between the two groups in CNO and CMO, which were defined as the main outcomes. Birthweight and GAB were identified as independent risk factors for predicting CMO {[odds ratio (OR)=1.273, 95% confidence interval (CI) 1.063–1.524, p=0.009]} and CNO (OR=0.606, CI 0.494–0.744, p<0.001) (Table 3). In this context, each 100-g increase in birthweight contributed to a 1.273-fold increase in the adverse consequences of CMO, whereas each 1-week increase in GAB contributed to a 1.650-fold protection against the adverse consequences of CNO.

**Table 2:** Birth characteristics and early neonatal and maternal outcomes of the study groups.

Variables		Group 1 (n=258)	Group 2 (n=35)	Total (n=293)	p-value
Cesarean delivery		175 (67.8)	27 (77.1)	202 (68.9)	0.356
GAB (weeks)		36 (34–38)	34 (31.25–35)	36 (33–38)	**<0.001**
PTB		32 (12.4%)	7 (20%)	39 (13.3%)	0.285
	<34 weeks	4 (12.5%)	1 (14.3%)	5 (12.8%)	>0.05
	≥34 weeks	28 (87.5%)	6 (85.7%)	34 (87.2%)	
Birthweight (g)		3,465 (3,205–3,755)	3,605 (3,300–3,960)	3,490 (3,212.5–3,790)	0.085
APGAR 1		9 (9–9)	9 (9–9)	9 (9–9)	0.325
APGAR 5		10 (10–10)	10 (10–10)	10 (10–10)	0.363
Female gender		106 (41.1)	15 (42.9)	121 (42.3)	0.987
NICU admission		21 (8.1)	5 (14.3)	26 (8.9)	0.215
Hypoglycemia		0 (0)	2 (5.7)	2 (0.7)	**0.014**
Hyperbilirubinemia		4 (1.6)	1 (2.9)	5 (1.7)	0.473
RDS		9 (3.5)	2 (5.7)	11 (3.8)	0.627
CNO		22 (8.5%)	5 (14.3%)	27 (9.2%)	0.344
PROM		16 (6%)	1 (2.9%)	17 (5.6%)	0.704
HDP		3 (1.2%)	1 (2.9%)	4 (1.4%)	0.400
PPH		4 (1.6%)	1 (2.9%)	5 (1.7)	0.473
CMO		21 (8.1%)	2 (5.7%)	23 (7.8%)	1.000

APGAR: appearance, pulse, grimace, activity, and respiration score; CMO: composite maternal outcome; CNO: composite neonatal outcome; GAB: gestational age at birth; HDP: hypertensive disorders in pregnancy; NICU: neonatal intensive care unit; PHA: polyhydramnios; PPH: postpartum hemorrhage; PROM: premature rupture of membranes; PTB: preterm birth; RDS: respiratory distress syndrome. Data are expressed as median (Q1–Q3) or number (percentage) where appropriate. A p<0.05 indicates a significant difference. Statistically significant p-values are indicated in bold.

**Table 3:** Multivariate logistic regression analysis of risk factors for composite maternal and composite neonatal outcomes.

Variables	Composite maternal outcome		Composite neonatal outcome	
	OR (95%CI)	p-value	OR (95%CI)	p-value
Age	0.954 (0.815–1.118)	0.563	1.057 (0.983–1.137)	0.132
BMI	0.801 (0.641–1.001)	0.051	1.041 (0.948–1.143)	0.400
Birthweight (100 g)	1.273 (1.063–1.524)	**0.009**	0.991 (0.905–1.084)	0.837
GAB	0.934 (0.548–1.593)	0.803	0.606 (0.494–0.744)	**<0.001**

BMI: body mass index; CI: confidence interval; GAB: gestational age at birth; OR: odds ratio. Data are expressed as median (minimum–maximum). A p<0.05 indicates a significant difference. Statistically significant p-values are in bold.

## DISCUSSION

The main findings were as follows. (1) The rates of adverse CNO, CMO, or any of the individual maternal and neonatal complications were similar in both groups, whereas neonatal hypoglycemia was more common in the polyhydramnios due to late-onset GDM group. (2) GAB was significantly lower in the polyhydramnios due to late-onset GDM group. Therefore, the higher rates of neonatal hypoglycemia (0 versus 2 neonates) could be related to the higher rate of preterm birth in this group. (3) Cesarean delivery rates were high in both groups compared with the general population, but no significant difference was observed between the two groups. (4) Birthweight and GAB were identified as independent protective factors predicting CMO and CNO, respectively.

In a retrospective study, abnormal results were obtained after rescreening with a glucose challenge test (GCT) in 165 of 513 women with previously normal GCT results who had at least one of the risk factors for GDM, such as obesity, hypertension, and family history of diabetes. Of these women, 154 underwent OGTT, and 20 of them were eventually diagnosed with late-onset GDM. Among the risk factors for GDM, only age greater than 30 years was significantly associated with the diagnosis of late-onset GDM, while there were no analyses of pregnancy outcomes, perinatal outcomes, and neonatal outcomes. Consequently, rescreening for GDM was recommended in advanced maternal age and in pregnancies with macrosomia, even if previous screening tests were negative^11^.

In a recent study by Parveen et al, 71 women diagnosed with early-onset GDM by OGTT performed before 24 WG due to the presence of risk factors for GDM were compared with 90 women diagnosed with GDM at or after 24 WG. A history of GDM, macrosomia, and stillbirths in previous pregnancies were found to be significant risk factors for predicting early-onset GDM. Early-onset GDM significantly increased rates of recurrent urinary tract infection, polyhydramnios, intrauterine fetal loss, mac- rosomia, fetal birth trauma and related conditions, low APGAR scores, and NICU admission, and it was significantly related to decreased fetal movements, possibly due to polyhydramnios^12^. These results were confirmed in other studies^13,14^. Therefore, in women with risk factors for developing GDM, diagnosis by OGTT in early pregnancy can lead to a significant reduction in poor pregnancy outcomes for both the mother and the fetus.

In another recent retrospective study by Cauldwell et al, pregnant women diagnosed with late-onset GDM by home glucose monitoring at or after 33 WG had higher rates of macrosomia and associated shoulder dystocia, PPH, third- and fourth-degree vaginal injuries, and NICU admissions than pregnant women without GDM and/or with early-onset GDM diagnosed by OGTT before 33 WG^15^. This study concluded that active GDM management can improve perinatal outcomes in all pregnant women with risk factors for late-onset GDM, regardless of specific glucose thresholds. However, in contrast to our study, women with late-onset GDM were not screened for GDM at or before 24–28 WG. Therefore, the high complication rates in these pregnancies are likely due to overlooked diagnosis of GDM (delayed diagnosis of GDM) in the early stages of pregnancy and prolonged exposure of the fetuses to a hyperglycemic environment. Furthermore, a 75-g OGTT performed between 24 and 28 weeks were normal in all participants in our study, so all patients diagnosed with late-onset GDM had actual late-onset GDM. Therefore, the duration of fetal exposure to a hyperglycemic environment is limited in this group, explaining that late development of GDM has less impact on fetal weight gain and that resulting macrosomia is not common. Accordingly, there were no significant differences in most pregnancy outcomes between patients with isolated polyhydramnios and patients with polyhydramnios due to late-onset GDM. In parallel with our study, Sohn et al found no difference in perinatal outcomes between patients without GDM and those diagnosed with late-onset GDM after an OGTT performed for suspected large-for-gestational-age fetus at an advanced stage of their pregnancy, despite normal GDM screening results in their early pregnancy^16^.

Among the recommended laboratory tests to be performed when investigating the etiology of polyhydramnios, a 75-g OGTT ranks first to rule out GDM^7^. However, pregnancy outcomes are similar in women with isolated polyhydramnios and with polyhydramnios due to late-onset GDM, identifying GDM as the cause of third-trimester polyhydramnios that is not of fetal–placental origin and repeating an OGTT despite a previous negative OGTT not only has no impact on pregnancy management but also may result in additional health care costs and unnecessary parental worry.

Our study has some limitations, mainly owing to its retrospective design and relatively small sample. The major strength of this study is the ability to analyze a specific population that has not been thoroughly studied on this topic. In addition, the study was conducted in a single tertiary medical center with a high volume of patients, where the standardized algorithms for diagnosis, treatment, and follow-up of polyhydramnios and GDM were applied.

## CONCLUSION

Polyhydramnios after the second trimester without evidence of fetal–placental causes leading to polyhydramnios may be a sign of late-onset GDM in women with previously normal OGTT results. As our study showed no difference in pregnancy outcomes and management was indifferent between isolated polyhydramnios and polyhydramnios due to late-onset GDM, it does not seem necessary or useful to identify late-onset GDM in patients with polyhydramnios in the third trimester. We think our study will guide further prospective randomized controlled trials to draw more definitive conclusions on this topic.
